# Case series: coronavirus disease 2019 infection as a precipitant of atypical hemolytic uremic syndrome: two case reports

**DOI:** 10.1186/s13256-021-03144-2

**Published:** 2021-12-13

**Authors:** Christine J. Kurian, Zachary French, Patrick Kukulich, Matthew Lankiewicz, Sushil Ghimire, Omar H. Maarouf, Sanaa Rizk, Ruben Rhoades

**Affiliations:** 1 Department of Medicine, Thomas Jefferson University, Philadelphia, PA USA; 2 Sidney Kimmel Medical College, Thomas Jefferson University, Philadelphia, PA USA; 3 Department of Medical Oncology, Thomas Jefferson University, Philadelphia, PA USA; 4 Department of Medicine, Division of Nephrology, Thomas Jefferson University, Philadelphia, PA USA; 5 Department of Medicine, Division of Hematology, Thomas Jefferson University, Philadelphia, PA USA

**Keywords:** Atypical hemolytic uremic syndrome, COVID-19, Nonimmune hemolytic anemia, SARS-CoV-2, Thrombotic microangiopathy

## Abstract

**Background:**

Atypical hemolytic uremic syndrome is an exceedingly rare thrombotic microangiopathy caused by accelerated activation of the alternative complement pathway.

**Case presentation:**

Here, we report two cases of patients presenting with suspected atypical hemolytic uremic syndrome precipitated by coronavirus disease 2019 infection. The first patient, a 25-year-old Hispanic male, had one prior episode of thrombotic microangiopathy presumed to be atypical hemolytic uremic syndrome precipitated by influenza A, and re-presented with thrombocytopenia, microangiopathic hemolytic anemia, nonoliguric renal failure, and normal ADAMTS13 activity, with confirmed coronavirus disease 2019 positivity. The second patient, a 31-year-old Caucasian female, had no personal history of thrombotic microangiopathy, though reported a family history of suspected atypical hemolytic uremic syndrome. She presented with similar laboratory derangements, oliguric renal failure requiring hemodialysis, and confirmed coronavirus disease 2019 positivity. Both patients were treated with eculizumab with complete resolution of their hematologic and renal complications.

**Conclusion:**

To our knowledge, this represents the largest case series of atypical hemolytic uremic syndrome precipitated by coronavirus disease 2019 in adults.

## Background

Hemolytic uremic syndrome (HUS) is defined by the presence of microangiopathic hemolytic anemia, thrombocytopenia, and renal impairment, and is frequently seen in children [1, 2]. The hallmark of atypical HUS (aHUS) is dysfunction of the complement system causing impairment of complement regulatory proteins and thus an accelerated activation of the complement pathway. aHUS is rare, with an incidence of 1 in 500,000 people per year in the USA [3]. The underlying causes of aHUS have been widely theorized and include both genetic and environmental factors. Genetic mutations in the genes *C3*, membrane cofactor protein (*CD46*), *CFB*, complement factor H (*CFH*), *CFHR1*, *CFHR3*, *CFHR4*, complement factor I (*CFI*), *DGKE*, and *THBD* are thought to contribute to 60% of cases [4–6]. A genetic predisposition coupled with an inciting event can result in an episode of aHUS [7]. These triggers can include infection, chemotherapy, immunotherapy, antiplatelet agents, malignancy, stem cell or solid organ transplantation, pregnancy, and underlying autoimmune conditions [4]. Here, we present details of two cases of aHUS in adults that were precipitated by coronavirus disease 2019 (COVID-19).

## Case 1

A 25-year-old Hispanic male with a recent history of thrombotic microangiopathy (TMA) (presumed influenza A-HUS) in January 2020 presented again in May 2020 with symptoms of fever, malaise, and nausea but without upper respiratory symptoms, cough, shortness of breath, or supplemental oxygen requirement. He tested positive for COVID-19.

**Fig. 1:**
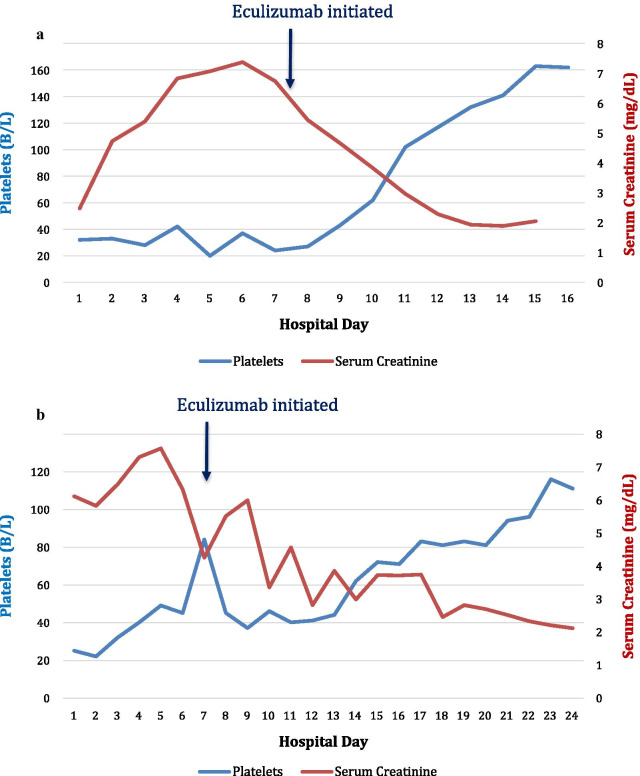
Lab trends in relation to eculizumab initiation for both patients. **a** Trend of platelet count (B/L) and serum creatinine (mg/dL) during case 1 hospitalization, with note of day of eculizumab initiation. On eculizumab initiation, platelet count was 27 B/L and creatinine was 5.44 mg/dL. **b** Trend of platelet count (B/L) and serum creatinine (mg/dL) during case 2 hospitalization, with note of day of eculizumab initiation. On eculizumab initiation, platelet count was 84 B/L and creatinine was 4.25 mg/dL

In January 2020, he had presented with fevers, nausea, and malaise and was diagnosed with influenza A via polymerase chain reaction (PCR). Additionally, he had acute kidney injury, hemolytic anemia, and thrombocytopenia suggesting TMA [thrombotic thrombocytopenic purpura (TTP) versus HUS/aHUS] triggered by infection. He was treated empirically with corticosteroids and seven sessions of therapeutic plasma exchange (TPE) without significant improvement. ADAMTS13 activity was measured but only after a partial session of plasma exchange and was > 100%. In the absence of other clear etiologies, influenza A-HUS was the presumed diagnosis. Following cessation of TPE, his thrombocytopenia improved to 162 B/L and his renal function plateaued at a serum creatinine (Cr) of 2.22 mg/dL (Fig. 1a). He was discharged after 8 days.

On his second presentation in May 2020, his hemoglobin (Hgb) was 13.7 g/dL but peripheral smear showed two to three schistocytes per high-power field (hpf), platelet count of 32 B/L, lactate dehydrogenase (LDH) of 1486 U/L, haptoglobin < 10 mg/dL, and indirect bilirubin of 1.9 mg/dL. Viral studies [human immunodeficiency virus (HIV), hepatitis B virus (HBV), hepatitis C virus (HCV)] were negative. His complement levels were notable for a decreased C3 serum level (72 mg/dL; normal range 88–201 mg/dL), and normal C4 serum level (20 mg/dL; normal range 10–44 mg/dL). Prothrombin time (PT)/international normalized ratio (INR) and partial thromboplastin time (PTT) were normal, and direct antiglobulin test was negative. Cr was 2.48 mg/dL (baseline ~ 2.2–2.4 mg/dL from the prior hospitalization in January 2020).

ADAMTS13 activity was determined prior to starting TPE. When ADAMTS13 activity was normal at 97% on day 5, TPE was stopped. His platelets had not improved, and his renal failure continued to worsen with a Cr peak at 7.38 mg/dL. With a high degree of suspicion for aHUS, he was then started on eculizumab (900 mg dose) on day 5 of hospitalization. After initiation of eculizumab, his thrombocytopenia and renal function began to improve (see Fig. 1a). He did not require renal replacement therapy. Another dose of eculizumab was given on day 12 of hospitalization. The patient was discharged that same day, and further treatment with eculizumab continued as an outpatient. Two weeks after discharge, his Cr normalized to 1.01 mg/dL and platelets were 185 B/L. His Hgb normalized within 3 months. His hematologic and renal parameters remained normal at 6-month follow-up with Hgb 14.0 g/dL, platelets 255 B/L, and Cr 0.97 mg/dL. He is currently being transitioned to ravulizumab for maintenance therapy, and has received appropriate vaccinations and antibiotic prophylaxis. As of June 2021, his most recent creatinine is 0.78 mg/dL and platelets are 203 B/L.

## Case 2

A 31-year-old healthy Caucasian female presented to the emergency department (ED) with a 10-day history of generalized fatigue, headache, chills, nausea, nonbloody diarrhea, and decreased urine output with dark-colored urine. She denied having fevers, cough, shortness of breath, dysuria, or hematuria. She did not take any prescription or over-the-counter medications. Notably, she has a family history of HUS involving two paternal uncles.

On arrival to our institution, initial labs were notable for Hgb 7.1 g/dL, platelets 25 B/L, Cr 6.11 mg/dL, haptoglobin < 10 mg/dL, LDH 1834 IU/dL, and total bilirubin 1.7 mg/dL. Peripheral smear confirmed presence of schistocytes at 5–10 per hpf. ADAMTS13 activity, inhibitor, and aHUS complement panel were sent, and she was initiated on corticosteroids and TPE. Her d-dimer levels were elevated at 2153 ng/dL, but ferritin was normal at 325 ng/mL. Her complement C3 level was mildly depressed at 84 mg/dL.

She had minimal response to TPE and corticosteroid treatment. On day 3 of admission, ADAMTS13 activity was 119% with inhibitor level < 0.4 units; thus, steroids and TPE were discontinued. Stool was negative for Shiga toxin-producing *Escherichia coli* (STEC). Given the reported family history of HUS, aHUS was high on the differential. Due to progressive oliguric acute kidney injury, she was initiated on hemodialysis on day 5 of hospitalization. The patient was initiated on eculizumab on day 6, with a dose of 900 mg intravenously weekly. The results of aHUS complement panel showed a normal C3 level, complement factor I, factor B, and factor H but with low C4 at 5.7 mg/dL and a significantly elevated factor H autoantibody at 238 units/mL (reference level < 22 U/mL). She had a gradual response to eculizumab, and by day 11 of her first eculizumab dose she was able to come off dialysis and her platelets had improved to 81 B/L and Hgb to 8.4 g/dL (see Fig. 1b for trend of Cr and platelet levels). Repeat COVID-19 testing on day 10 of hospitalization remained positive, although she never had respiratory complaints or required supplemental oxygen. Her clinical picture suggested aHUS triggered by COVID-19 infection. Given the response to eculizumab, the decision was made to continue outpatient eculizumab. Her last laboratory results 3 months after initial diagnosis showed normal Hgb at 12.3 g/dL, platelet counts of 171 B/L, and Cr of 1.1 mg/dL. She remains dialysis-independent and on maintenance eculizumab every 2 weeks, with appropriate vaccinations and antibiotic prophylaxis. As of August 2021, her most recent creatinine is 1.1 mg/dL and platelets are 212 B/L.

## Discussion

Atypical HUS is a rare thrombotic microangiopathy most commonly presenting in children. The pathophysiology is complex but is largely attributed to accelerated activation of the alternative complement system leading to endothelial dysfunction and subsequent thrombotic microangiopathy with end-organ damage. This can occur either by inherited mutations in the complement regulatory genes or by acquired autoantibodies to complement regulatory proteins, most commonly against complement factor H.

To our knowledge, this is the first series of adults with aHUS associated with/precipitated by COVID-19 infection. The patient in case 1 posed a complicated diagnostic dilemma. Given the clinical picture including COVID-19, depressed C3 complement level, acute kidney injury, normal ADAMTS13 level, and response to eculizumab, aHUS triggered by COVID-19 was the most likely diagnosis.

Since the episodes of recurrent TMA were triggered by different viral illnesses (influenza A and COVID-19), it is plausible that he has an underlying genetic cause with mutations in genes regulating complement pathway as discussed above.

In contrast, the patient in case 2 had a family history significant for HUS, normal ADAMTS13 activity and stool infectious studies, a high titer of anti-CFH autoantibody, and slow but excellent response to eculizumab, all of which favor a diagnosis of aHUS, with likely COVID-19 trigger. The anti-CFH antibodies interfere with factor H binding to the alternate pathway C3 convertase, thus causing a defect in factor H-dependent cell protection. More than 90% patients with anti-CFH autoantibodies also are found to have a deletion in *CHFR1* and *CHFR3* genes [8]. Thus, an underlying genetic cause is still possible and may be explored despite positive antibody titers.

Despite the lack of genetic testing in both of our patients, which would provide additional evidence for their diagnoses, the exclusion of alternative diagnoses as well as the marked improvement with eculizumab make aHUS triggered by COVID-19 most likely. Remarkably, both patients’ kidney function and platelets normalized with eculizumab treatment.

The mechanism of aHUS precipitated by COVID-19 is unknown, although multiple theories have been proposed. Direct endothelial damage by COVID-19 may be a contributing mechanism to its precipitation of aHUS. One case report notes severe endothelial injury in lung tissue of seven patients who died from COVID-19, which was unique when compared with lungs from patients with influenza A or uninfected controls [9]. Another report proposes the mechanism of endotheliitis by COVID-19 being not only due to direct effects of viral involvement and the subsequent inflammatory response, but also via induction of apoptosis and pyroptosis [10]. A potential mechanism for this may be binding to the severe acute respiratory syndrome coronavirus 2 (SARS-CoV-2) receptor, angiotensin-converting enzyme 2 (ACE2), which is highly expressed on vascular endothelial cells [11, 12]. Complement activation by COVID-19 is another leading theory regarding the mechanism of aHUS precipitated by COVID-19. For example, one case report detailed the findings that lung and skin tissue of patients with severe COVID-19 infection with either respiratory failure or purpuric skin rash had substantial deposits of complement cascade proteins within the microvasculature, suggesting a systemic activation of the complement pathways by COVID-19 [13]. Lastly, recent evidence suggests the spike protein may directly activate the alternative complement pathway [14]. This is supported by a recent report of four COVID-19 patients being successfully treated with eculizumab for severe pneumonia and acute respiratory distress syndrome (ARDS) [15].

With the advent of vaccines, the landscape of the COVID crisis is changing rapidly. These patients were encouraged to receive a COVID-19 vaccine but were counseled that their immune response may be suboptimal while on terminal complement blockade therapy. The patient in case 1 has not yet been vaccinated, but has plans to do so as of August 2021. The patient in case 2 received two doses of the Moderna vaccine in January 2021. Antibody titers checked in August 2021 were strongly positive.

## Conclusion

Atypical HUS is a rare diagnosis with high morbidity and a significant number of patients needing dialysis, mainly due to a delay in diagnosis. Timely diagnosis and initiation of treatment with eculizumab is of utmost importance and can help reduce the need for dialysis, permanent renal injury, and even death. With the ongoing pandemic, COVID-19-triggered aHUS should be kept in differential and early institution of eculizumab therapy should be considered to improve patient outcomes.

## Data Availability

Data sharing is not applicable to this article as no datasets were generated or analyzed during the current study

## Abbreviations

aHUS: Atypical hemolytic uremic syndrome
COVID-19: Coronavirus disease 2019
ARDS: Acute respiratory distress syndrome
